# COVID-19 Outcomes in Minority Ethnic Groups: Do Obesity and Metabolic Risk Play a Role?

**DOI:** 10.1007/s13679-021-00459-5

**Published:** 2021-10-15

**Authors:** Paul Coleman, Thomas M. Barber, Thijs van Rens, Petra Hanson, Alice Coffey, Oyinlola Oyebode

**Affiliations:** 1 Warwick Medical School, University of Warwick, Coventry, UK; 2 Department of Economics, University of Warwick, Coventry, UK

**Keywords:** COVID-19, Ethnic inequalities, Obesity, Metabolic risk, Social determinants of health

## Abstract

***Purpose of Review*:**

Globally, minority ethnic groups have been at higher risk of COVID-19 mortality and morbidity than majority populations. This review outlines factors that may interact to create these inequalities and explores the hypothesis that differing levels of cardio-metabolic risk, according to ethnic group, play a role.

***Recent Findings*:**

Two UK Biobank studies have reported that the body mass index is more strongly associated with an increased risk of COVID-19 infection and mortality in minority ethnic populations than in White populations. A study of UK patients found that the strongest association between obesity and adverse COVID-19 outcomes was in people of Black ethnicity.

***Summary*:**

Differences in the prevalence of obesity and its metabolic sequelae have been shown to partly mediate ethnic inequalities in COVID-19 outcomes, although not always consistently. It is possible that ethnic differences in the consequences of obesity may explain some of the remaining disparity in COVID-19 risk.

## Introduction

Globally, minority ethnic groups have been at higher risk of COVID-19 mortality and morbidity than majority populations. This disparity likely arises from multiple factors that interact and manifest as both increased risk of infection and adverse clinical outcomes from infection. Some of the factors discussed in the media include differential exposure to the COVID-19 virus, confounding by socio-economic status, and institutional racism within the health care sector [1–3]. Furthermore, differences in the prevalence of certain comorbidities (such as Diabetes Mellitus and obesity), that contribute towards cardio-metabolic risk, and also increase the risk of COVID-19, have also been suggested to play a role. In this review, we outline why it is useful to explore the ethnic inequalities in COVID-19 outcomes and some of the factors that may play a role in creating these inequalities. We summarise evidence linking obesity and cardio-metabolic risk to COVID-19 outcomes, and explore the hypothesis that differing levels of cardio-metabolic risk according to ethnic group play a role in the poorer COVID-19-related outcomes experienced by minority ethnic groups.

## What is Ethnicity and Why Should We Study It?

The concept of ‘race’ provides a way of defining populations that look different and have different ancestral roots [4]. ‘Ethnicity’ is a multi-faceted quality that refers to the group to which people belong, or are perceived to belong, as a result of certain shared characteristics, including geographical and ancestral origins, but particularly cultural traditions and languages [5]. Ethnicity is self-defined [4]. Worldwide, the concept of ‘ethnicity’ is overtaking that of ‘race’ in epidemiological research.

Ethnicity and race are controversial concepts in research, with historical racialised research being used to justify slavery, imperialism, anti-immigration policy and the social status quo [6]. The study of race or ethnicity is difficult in many European countries due to restrictions in the collection of data on ‘racial or ethnic origins’ [7]. It is worth noting that genetic studies have revealed striking homogeneity amongst people who might be categorised as belonging to different ‘races’, and greater genetic heterogeneity amongst the populations living in Africa, then amongst the entire non-African human population [8]. It is also likely that the terms ‘race’ and ‘ethnicity’ will become less meaningful as international travel, migration and ‘inter-racial’ marriage continue a steep-upward trajectory [9]. However, analysing differences in the patterns of morbidity and mortality between populations and understanding why these arise is a key component of epidemiological research. These analyses provide an evidence base for public health policy and practice which seeks to mitigate preventable inequalities in the incidence and prevalence of disease between population groups, by addressing the key determinants of those inequalities.

There are known differences in health outcomes according to ethnic group [10]. Governments, policymakers and health professionals recognised early in the COVID-19 pandemic that ethnicity appeared to be associated with worse COVID-19 outcomes [11–13]. A clearer understanding of the differences in COVID-19 outcomes by ethnic group could provide valuable insights into our understanding of the transmission and clinical sequelae of this disease, ultimately to support improvements in future prevention and treatment strategies. Furthermore, investigation of inequalities in COVID-19 infection and outcomes illuminate processes of marginalisation relevant to the experience of minority ethnic groups more generally [14].

## Difference in COVID-19 Outcomes by Ethnicity

There are differences in vulnerability to COVID-19 between ethnic groups reported in numerous studies. In the USA, analysis of health service data from one of the largest health record providers, incorporating data from 50 million active patients whose ethnicity data were recorded, found that people from minority ethnic groups were more likely to be positive when tested and to require a higher level of care at the time they tested positive for COVID-19. The same study found that Black, Hispanic and Asian patients who had tested positive for COVID-19 had significantly higher rates of hospitalisation and death compared to their White counterparts and that differences in hospitalisation and mortality persisted even after controlling for socio-demographic factors and comorbidities [15].

In the UK, a prospective cohort study of over 30,000 patients found that people belonging to minority ethnic groups in hospital with COVID-19 were more likely to be admitted to critical care and receive invasive medical ventilation than White patients. This was despite similar disease severity on admission and similar duration of symptoms, and after adjusting for age, sex, location, deprivation and comorbidities [16]. Other analyses of UK data report similar findings [17, 18]. In Brazil, a study of just under 100,000 patients found that, compared with White Brazilians (the majority population), Black Brazilians and Pardo Brazilians (those with mixed heritage) had significantly higher mortality [19].

As discussed above, there are many European countries in which analysis by ethnic group is not possible due to restrictions on the collection of data. However, migrant status is often recorded, allowing identification of those born in another country. Migrants form a smaller and separate group that likely overlaps considerably with minority ethnic groups. A systematic review and meta-analysis of COVID-19-related outcomes in migrants to high-income countries identified, appraised and synthesised 59 studies from Sweden, Italy, the USA, Canada, the Netherlands, Greece, Saudi Arabia and across the EU/EEA and UK, concluding that migrants account for a disproportionately large number of COVID-19 infections and deaths. This is despite relatively low rates of testing for COVID-19 infections in the migrant population [20].

## Possible Contributing Factors to Ethnic Inequalities in COVID-19 Outcomes

**Fig. 1:**
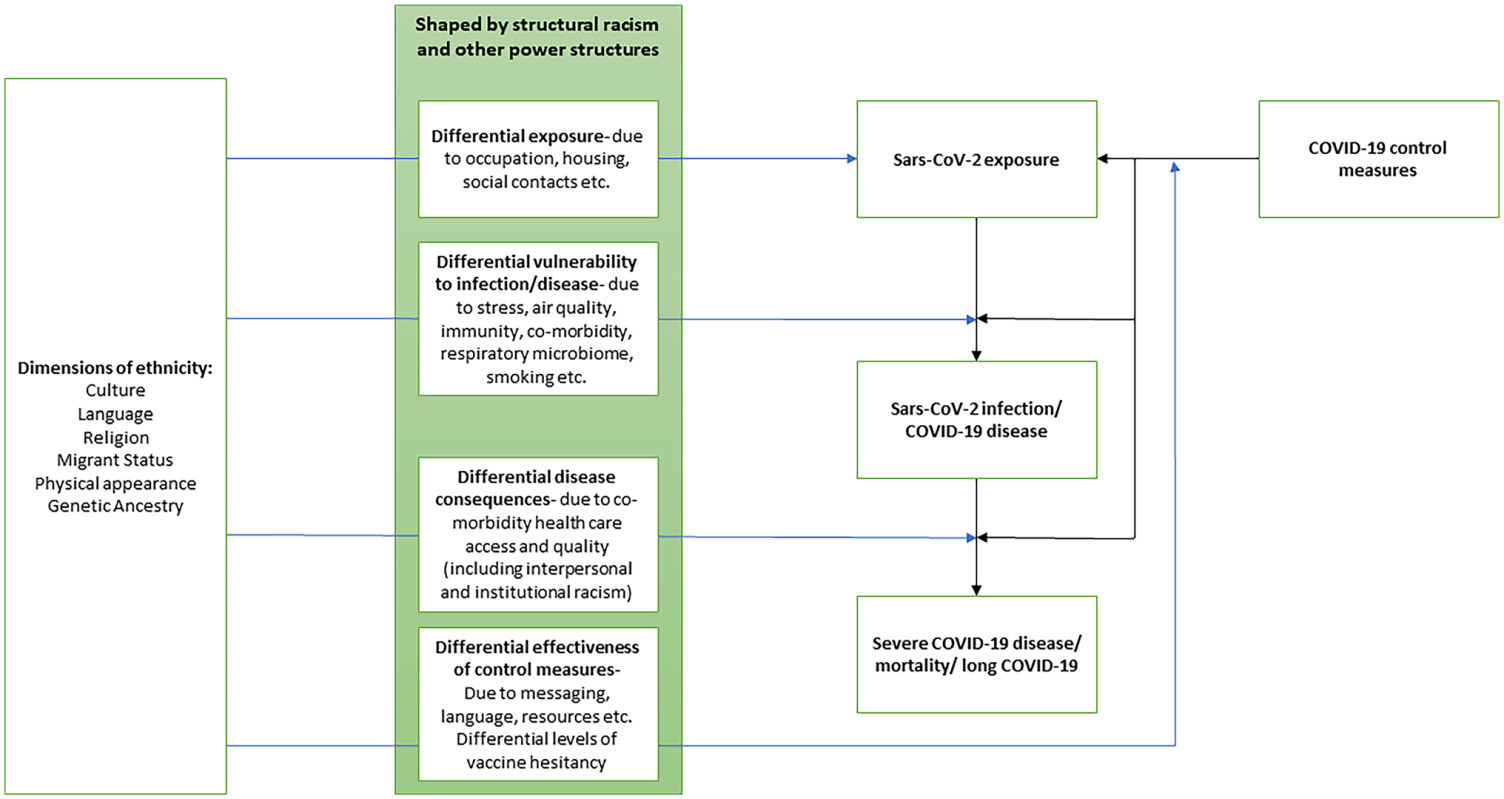
Multiple interacting factors with a role in differential COVID-19 outcomes according to ethnicity: adapted from a framework for understanding pathways underpinning ethnic inequalities in COVID-19 and potential targets for policy by Srinivasa Vittal Katikireddi et al. K Epideimiol Community Health https://doi.org/10.1136/jech-2020-216061

Numerous factors have been put forward to explain the ethnic inequalities in COVID-19-related outcomes. We know that obesity appears to moderate vulnerability to COVID-19 and we know that obesity and its sequelae affect different ethnic groups differently; however, this is just one of several possible hypotheses for the differences in COVID-19 outcomes. Before considering the role that obesity and cardio-metabolic risk may play (in ‘Testing the Hypothesis That Differing Cardio-Metabolic Risk Plays a Role’), we outline the other various factors that are theoretically and empirically reported to play a role in the observed inequalities to put ‘Testing the Hypothesis That Differing Cardio-Metabolic Risk Plays a Role’ in context. We will use part of a published framework for understanding the pathways that may have generated inequalities in COVID-19 (Fig. 1) [21•]. This was developed from a well-established framework for studying health inequalities [22, 23]. We focus on exposure, infection and disease outcomes from COVID-19 infection rather than the social consequences of disease.

According to this framework, those factors theoretically contributing to the differential health consequences of COVID-19 include:

### Differential Exposure (due to Occupation, Housing, Social Contacts, etc.)

Various studies have reported that differential occupations, area of residence (more minority ethnic groups in urban areas where transmission occurs at a higher rate), living arrangements (size and composition of households) and increased reliance on public transport partly explain observed inequalities in COVID-19-related outcomes by ethnic group [18, 24, 25].

### Differential Vulnerability to Infection/Disease (due to Stress, Immunity, Smoking, etc.)

Differential exposure to the virus might underlie differences in the rate of infection between ethnic groups, but it is also possible that minority ethnic groups are more likely to be infected when exposed. Increased prevalence of vitamin D deficiency in minority ethnic groups (likely due to darker skin colour and cultural differences in terms of clothing coverage) has been suggested as important in mediating possible reduced immune response to COVID-19. Although there is no evidence for this with respect to COVID-19, a meta-analysis of 25 randomised controlled trials has demonstrated that vitamin D supplementation protects against other viruses that cause acute respiratory illnesses [26].

Another possible mediator of differential vulnerability is air pollution. More minority ethnic groups live in urban areas, and high air pollution has been associated with higher COVID-19 mortality [27].

### Differential Disease Consequences (due to Comorbidity, Health Care Access and Quality (Including Interpersonal and Institutional Racism))

Potential reasons for more severe COVID-19 disease course in patients from minority ethnic groups include a difference in overall health status due to comorbidities, and differences in health care access and quality of care provided. We explore the role of obesity and cardio-metabolic co-morbidities in ‘Testing the Hypothesis That Differing Cardio-Metabolic Risk Plays a Role’, but here it is worth noting that in a UK analysis, patients from minority ethnic groups were less likely to have some comorbidities such as heart disease and dementia [16].

In terms of health care access and quality, later presentation of minority ethnic groups to health services and lack of access to care across many conditions have been consistently reported across many studies [28–31]. Differences in patient experience for those from minority ethnic groups have also been demonstrated [32–34]. There may be specific financial and structural barriers to health care access by migrants; for example in the USA, migrants may lack health insurance [35]. With respect to COVID-19, a US study of data from 50 million patients found that people from minority ethnic groups did not have higher COVID-19 testing rates compared to the majority White population despite higher positivity rates and need for a higher level of care on diagnosis [15]. Another study using the US National Health Interview Survey found that affordability of medicine was a significant predictor of severe COVID-19 risk for Black and Hispanic participants [36]. This evidence highlights that reduced access to health care for minority ethnic groups in the USA might increase the risk of poor COVID outcomes (for example, because patients are sicker when they do reach care). The quality of care patients receive when they access health services may also be affected by their ethnicity [37]. In combination, lack of access and lower quality of care could be responsible for poorer COVID-19 outcomes.

### Differential Effectiveness of Control Measures (due to Messaging, Language, Resources, etc.)

There is some evidence that limited access to culturally appropriate information or language barriers might result in less knowledge regarding transmission, symptoms and appropriate preventative actions in some minority ethnic groups [38]. Vaccine hesitancy in the UK is higher amongst Black and Pakistani/Bangladeshi ethnic groups [39]. This may be explained by differences in socio-economic status, and it is worth noting that pre-pandemic studies do not consistently show vaccine hesitancy amongst minority ethnic groups [40]. Alternatively, vaccine hesitancy might reflect lower trust in authorities that is specific to minority ethnic groups and related to experiences of discrimination.

If control measures are less effective for those from minority ethnic groups, this augments any differences in exposure to the virus. If vaccine hesitancy translates into reduced vaccine take-up, it may mean COVID-19 continues to be a risk for some ethnic groups for some time.

### Wider Context

The factors outlined in this section are influenced by structural racism and other power structures that interact with the wider social determinants of health. Having outlined the multiple interacting factors contributing to differential experience of COVID-19 according to ethnic group, in the rest of this paper we explore one of the comorbidities discussed in ‘Differential Disease Consequences (due to Comorbidity, Health Care Access and Quality (Including Interpersonal and Institutional Racism))’ above, in more detail: metabolic risk and in particular overweight and obesity. The next three sections describe why we may expect metabolic risk to be a relevant factor in the link between ethnicity and COVID-19 outcomes.

## The Role of Obesity and Cardio-Metabolic Risk in the COVID-19 Course

Obesity appears to be an important risk factor for both contracting COVID-19 and poorer clinical outcomes in COVID-19 patients. Analysis of the UK Biobank (a large prospective cohort of half a million adults) found that both body mass index (BMI) and waist circumference were associated with testing positive for COVID-19 in a dose–response fashion. Adjustment for possible confounders did not change the results [41]. A meta-analysis of 41 studies published up to July 2020 concluded that obesity was associated with an increased likelihood of a positive COVID-19 test and hospitalisation with COVID-19. Hospitalised patients with obesity were more like to be admitted to intensive care and to require invasive mechanical ventilation, and they were more likely to die in hospital. A higher severity of obesity was also associated with a greater risk of poor clinical outcomes from COVID-19 infection [42].

Obesity likely increases risk of poor COVID-19 outcomes through its association with cardio-metabolic risk factors (such as type 2 diabetes mellitus, hypertension and dyslipidaemia). Examination of the association between COVID-19 co-morbidities and mortality rate in a meta-analysis of 87 studies found that type II diabetes was the strongest predictor of mortality [43•]. A further meta-analysis of three studies published up to November 2020 found that COVID-19 patients requiring intensive care or invasive mechanical ventilation had higher visceral fat volumes than those patients without the need for these interventions [44]. Furthermore, in primary studies, hyperglycaemia at hospital admission associates with poor patient outcomes from COVID-19 infection, and in a large population-based study including patients with both type 1 and type 2 diabetes, COVID-19-related mortality was associated with cardiovascular complications, poor glycaemic control and a higher BMI [45, 46]. Hypertension, another common metabolic complication in people living with obesity, has been associated with more severe disease [47].

The underlying pathophysiological processes of obesity could explain the susceptibility for increased risk of severe disease. The chronic inflammatory processes induced by obesity are associated with immune dysfunction [48]. Furthermore, obesity leads to changes in the pulmonary mechanics and creation of ventilation-perfusion mismatch [49] as well as increasing the number of ACE2 receptors that enable entry of the virus to the cell [50, 51].

## Ethnic Differences in Cardio-Metabolic Risk

Cardio-metabolic risk factors vary across ethnic groups for a variety of reasons that may include genetic and epigenetic factors, as well as body size preferences, socio-economic factors and stress exposure [52]. Prevalence of obesity, defined as BMI ≥ 30 kg/m^2^, is higher in many minority ethnic groups than in majority populations [53, 54]. However, this BMI threshold, set in 1993, was based on observational studies of almost exclusively White populations. There is growing evidence that members of Black and Asian ethnic groups are likely to have increased cardio-metabolic risk compared to those from a White background at the same BMI. A recent large population-based study of electronic health records from more than a million people found that Black Caribbean, South Asian, Chinese and Arab populations living in England had an equivalent risk of type 2 diabetes at substantially lower BMI values than the current BMI cut-offs used to categorise obesity [55•]. This analysis suggested that for an equivalent age-adjusted and sex-adjusted incidence of type 2 diabetes at a BMI of 30 kg/m^2^ in White populations, population thresholds should be 23.9, 28.1, 26.9 and 26.6 kg/m^2^ for South Asian, Black, Chinese and Arab populations, respectively.

## Testing the Hypothesis That Differing Cardio-Metabolic Risk Plays a Role

Differences in the prevalence of obesity and its metabolic sequelae have been shown to partly mediate some of the differences in COVID-19 outcome by ethnic group. For example, a study of 35,000 hospitalised patients found that higher prevalence of diabetes mellitus explains some of the greater risk of dying for hospitalised patients from South Asian background in the UK, with mediation analysis in a survival model finding that 17.8% of the total effect of South Asian ethnicity on mortality was due to diabetes [16]. However, in another multivariable analysis of 30-day mortality in hospitalised COVID-19 patients, adjusted for age and sex, Asian or Asian British patients as well as Black or Black British patients had increased risk of death compared with White patients (hazard ratios of 1.49 (1.19–1.86) and 1.30 (1.02–1.65), respectively) which persisted after adjustment for comorbidities, specifically obesity (defined as BMI > 30), diabetes, hypertension and chronic kidney disease. Hazard ratios were barely changed after adjustment in this case (1.48 (1.09–2.01) and 1.32 (0.96–1.84), respectively) [17].

Other analyses that examine the differences in COVID-19 outcomes according to ethnic group have adjusted for comorbidities including obesity (by BMI or clinician-defined), diabetes and hypertension, with the reports generally suggesting that there may be attenuation of the inequalities, but that they still persist, for example, [15].

While higher prevalence of obesity and cardio-metabolic risk amongst minority ethnic groups may be part of the reason for poorer COVID-19 outcomes in these populations, it is possible that where this has not appeared to be the case—this is due to inappropriate thresholds being used to determine whether the participants have obesity or not. In the analysis of hospitalised patients referenced above, the BMI threshold of 30 kg/m^2^ has been used to define obesity across all ethnic groups [16]. Potentially, this underestimates the prevalence of obesity in minority ethnic groups, since lower thresholds may be more appropriate for these groups. It is also possible that, over and above differences in prevalence of obesity across ethnic groups, differences in the effect of obesity on metabolic function may explain some of the remaining difference in outcomes. That is, where adjusting for comorbidities does not completely attenuate differences between ethnic groups, increased metabolic dysfunction at comparatively lower BMIs may explain part of the remaining difference. There are only a few papers examining this possibility.

One of the existing studies used UK Biobank data linked to national COVID-19 laboratory test data up to 14th June 2020. This study found that BMI was associated with an increased risk of a positive test for COVID-19 in both minority ethnic and White individuals. However, the dose response differed by ethnic group. For those with a BMI value of 25 kg/m^2^, there was no difference in risk between those belonging to a minority ethnic group and White individuals (OR = 0.96; 95% CI: 0.61, 1.52). Conversely, for those with a BMI of 30 kg/m^2^ or 35 kg/m^2^, the odds of COVID-19 were 1.75 (1.24, 2.48) and 2.56 (1.63, 4.03) higher in members of minority ethnic groups compared with those for White individuals. This study is limited because testing during the time period (up to June 2020) was not extensive, and not random. However, these data do suggest ethnic differences in the way that obesity might influence vulnerability to contracting COVID-19 [56•]. A further analysis of UK Biobank data, which examined mortality as an outcome up to May 2020, found that BMI associated more strongly with COVID-19-related death in non-White than in White populations [57].

Finally, a study of 54,000 patients admitted to hospital in the UK with COVID-19 found that obesity was a risk factor for admission to critical care, mechanical ventilation and in-hospital mortality across all ethnic groups. However, the study further found that the strongest association between obesity and relevant outcomes was in people of Black ethnicity. The study concluded that for patients admitted to hospital, obesity is a stronger risk factor for Black ethnic groups specifically (but not for minority ethnic groups in general) [58•].

## Conclusion and Implications

Certainly, there is now much evidence from the UK and USA that minority ethnic groups are at increased risk of COVID-19 compared to majority populations, along with weighty evidence that migrant groups are at risk across many high-income countries, and some indication that minority ethnic groups in other countries are also at increased risk (specifically Brazil). While there is some evidence that greater prevalence of obesity and its sequelae in minority ethnic groups play a role in this increased risk, it is also theoretically possible that increased vulnerability to cardio-metabolic risk in some minority ethnic groups plays a role in this inequality. Evidence for this latter, disproportionate effect of obesity on COVID-19 outcomes in minority ethnic groups, is currently sparse.

Interdisciplinary research is likely to be required to maximise understanding of differences in COVID-19 outcomes between ethnic groups, as previously recommended by the UK’s Faculty of Public health [59]. Use of a framework, such as that developed by Katikireddi and colleagues, and used in our review may help researchers to consider the complex interplay of the wider social determinants of disease [21•].

Researchers looking at differences in COVID-19 outcome by ethnic group should consider that BMI may not indicate equivalent risk levels across groups, and could consider using ethnic-specific BMI thresholds or entering an interaction term for ethnicity and BMI, alongside terms for ethnicity and BMI in regression equations. An example of a regression equation that would give coefficients relating to (a) the effect of BMI on COVID-19 outcomes (including any effect relating to differing COVID-19 outcomes that is related to different ethnic groups having different mean BMI), (b) the effect of ethnicity on COVID-19 outcomes through channels other than differences BMI between groups and (c) the effect of ethnicity on COVID-19 outcomes due to BMI differentially impacting ethnic groups is shown in Box 1.

**Box 1:**
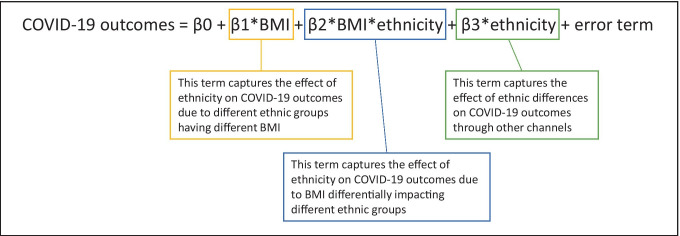
Researchers examining ethnic inequalities in COVID-19 outcomes using regression equations may wish to use an interaction term to capture the effect of BMI differentially impacting different ethnic groups

Further research could explore the size and direction of any increased risk by ethnic category in countries where the minority ethnic group is White (e.g. South Africa); or compare and contrast disease outcomes in the same ethnic group or a diaspora across multiple countries, including where they form a majority population; or look at risk in cohorts of migrants and their direct descendants. These analyses may be difficult to interpret, given the difference in contexts in different countries with varying levels of underlying comorbidities and very different health systems. However, it may be possible to tease apart the proportion of the difference in risk accounted for by some of the factors outlined in ‘Possible Contributing Factors to Ethnic Inequalities in COVID-19 Outcomes’ and Fig. 1.

For clinicians, the evidence reviewed here makes it clear that patients with obesity and those from minority ethnic groups with COVID-19 infection are at particularly high risk for adverse clinical outcomes. Given this evidence, it is incumbent upon all healthcare professionals to prioritise preventative management strategies for these vulnerable groups, including the focused promotion of COVID-19 vaccination. It may also be advisable to consider both whether a patient belongs to a minority ethnic group, as well as their degree of obesity, and co-presence of these risk factors in triaging decisions when prioritising rapid treatment.

The World Health Organisation capitalised on data showing the increased risk of COVID-19 for smokers, to promote smoking cessation to millions of tobacco users [60], and the UK prime minister reports his experience with COVID-19 as being a key motivator of his efforts to lose weight [61]. Primary care physicians who are the first point of contact with patient groups with obesity may wish to use similar strategies and messaging to support patients to start a journey towards weight loss.

For policymakers, the pressing health concern may be COVID-19, but it is worth noting that non-communicable disease risk factors interact with COVID-19 infection to bring about increased morbidity and mortality. For this reason, policymaking to reduce obesity at the population level must be maintained. In addition, given that the ethnic inequality here is well evidenced, a coherent plan on what to do about this inequality is required urgently. Medical and race equality organisations in the UK came up with a list of recommended actions one year ago, which may be a useful starting point [62]. Consideration of the best way to support dissemination of educational material and information might help to reduce inequalities. This might include targeting material specifically for ethnic minority groups (perhaps through engagement with places of worship or other community hubs) or creating campaigns which transcend language and cultural barriers.

Where socio-economic differences have been put forward as the main reason for ethnic inequalities in health (e.g., in [63]), this fails to recognise that structural and institutional racism is the main driver of these social factors, reinforcing existing inequalities. Working towards a society in which socio-economic inequalities between ethnic groups are extinguished is likely to make a large contribution to reducing ethnic inequalities in health— decreasing both inequalities in obesity prevalence and its sequelae, and in COVID-19 infection and outcomes.

## Data Availability

This review examines data and material from published literature.
